# Dr. Conolly on the Management of Lunatic Asylums

**Published:** 1848-01-01

**Authors:** 


Art. VII.—*The Construction and Government of Lunatic Asylums and Hospitals for the Insane.* By John Conolly, M.D., Physician to the Hanwell Asylum, Middlesex, &c. &c. London: Churchill, 1847.

The internal government and management of asylums for the cure and care of the insane! Can we conceive a subject of more importance than this, and one so specially fitted for the pen of the physician whose name stands at the head of this page? Dr. Conolly's well known ability, his acknowledged humanity, his great experience in the treatment of the insane, his long official association with one of the largest, and generally admitted to be amongst the best conducted, of our public asylums, peculiarly qualify him to speak *ex cathedra* on this subject, and to give to the profession and the public a correct idea of what such establishments should be.

These are points upon which experienced men can only speak satisfactorily. It is useless to theorize; the finest, the most plausible and philanthropic of our speculations, often signally fail when brought to the severe but proper test of practical observation. We therefore hail, with considerable satisfaction, the publication of Dr. Conolly's work on the Government of Lunatic Asylums. We know, and as we read page after page, we feel that we are rambling along (thanks to the benevolent spirits of the age!) a pleasant road, with an intelligent and agreeable traveller, who is acquainted, intimately acquainted, with every inch of ground over which we are passing.

Hanwell Asylum contains one thousand pauper patients! Let the hundreds who annually visit this noble institution, and wend their way through its wards, inspect its arrangements, and perambulate through its grounds, give evidence of the admirable skill with which everything is conducted. Dr. Conolly's spirit appears to pervade every department of the asylum; he is like a father among his children, speaking a word of comfort to one, cheering another, and exercising a kindly and humane influence over all; making the very atmosphere in which the patients live redolent of the best sympathies of our nature. He feels, as all ought to feel, who undertake the important, the anxious, and responsible management of the insane, *that the affliction of disease does not necessarily block up the avenues to the human heart;* that even in the worst, the most distressing forms of mental malady, there often exist some of the better principles of our spiritual being in all their original purity, upon which the physician and the moralist may act with advantage.

Wordsworth, the poet of nature, has beautifully delineated the influence of the kindly emotions of the heart upon persons gifted with healthy reasoning powers. How powerful is this appeal to those who have to do with the insane!

> "The poorest poor
> Long for a moment in a weary life,
> When they can know and feel that they have been
> Themselves the fathers, and the dealers out
> Of some small blessings; have been kind to such
> As needed kindness; for this single cause—
> *That we have all one human heart.*"

To fully appreciate this view of the case, it is necessary to live among the insane, to make them one's daily companions, and to become personally and practically acquainted with their habits and character of their minds. And how is it possible to carry out, with any prospect of a successful issue, anything like a connected, consecutive, and philosophic plan of medical or moral treatment, unless this course is pursued?

With regard to insanity, it should never be forgotten that the whole faculties of the mind are seldom implicated in the disorder. In affections of the intellect, of the reasoning powers, the feelings, the sentiments are often unaffected, and we have, therefore, a powerful moral fulcrum upon which to place our lever. In proportion to our ability to work upon the healthy characteristics of mind, shall we be better enabled to combat with the disordered perceptions and inclinations. If it be possible to educate idiots, as is successfully and conclusively established, or children almost imbecile, or of stunted intellectual growth, *à fortiori*, we are able, by well directed efforts, to teach persons palpably insane the habit of self-control, and to chase away from the brow of the invalid the furrowed lines of anxious thought, by patient and unwearied efforts to turn the mind into a healthy channel. We should never forget that insanity is not an affection of the mind *per se;* that to whatever amount of disturbance the intellect, sentiments, and affections may be subjected, it is nothing more than an effect or result of some impairment of the physical structure—the casket enclosing the precious jewel.

If we take this view of the rationale of mental derangement, we shall be enabled to form a proper estimate of the importance of working upon the mind itself, and gradually bringing it within the sphere of healthy influences. We feel confident that this mode of treatment is too often lost sight of. That we have it in our power to subdue abnormal conditions of mind, by conveying our remedial agents through mental channels, is undoubted. It is no argument against this view of the case to say, if the disorder of the understanding is consequent upon an alteration in the condition of the material organ of the mind, that we cannot get at the intellect except through physical media. We admit that we can only act upon mind through material channels. We obtain all our ideas: in fact, the mind is built up by means of *materials* obtained through the medium of our physical senses; therefore, when it is urged that it is impossible to work upon the diseased fancy without first influencing matter—we bow to the truism; but, nevertheless, this admission does not, in the slightest degree, militate against the importance—the great importance—of the view we have taken of the possibility of curing many forms of insanity by means strictly moral in their character. We do not conceive that this view of the treatment of certain states of mind is in opposition to our strongly expressed opinion of the marked and essential benefit which often accrues from a judicious administration of physical remedies. We are now referring to cases in which moral means are the great elements of cure—cases in which it is impossible to detect, after the most careful investigation, the slightest appreciable bodily ailment; but often, in such instances, the physical and moral treatment must proceed *pari passu*, and the skilful and experienced physician shows his knowledge of the malady, and his own ability, by wielding both these agents without appearing to use either.

Having made these preliminary observations, we now proceed to analyze fully the views taken by Dr. Conolly in the volume before us.

He commences his essay by some remarks on the necessity of erecting additional county lunatic asylums, and says, that, according to the Report of the Commissioners in Lunacy for 1844, the number of insane poor in England and Wales was 17,000, out of which no more than 4500 have any provision in public asylums! The act of parliament provides, however, for this calamity, but Dr. Conolly observes, that much opposition is made in several counties to the erection of additional asylums, owing to the idea entertained by a portion of the magistracy that pauper lunatics are not only more cheaply provided for in private licensed houses than in county asylums, but that the number of cures effected in private houses is greater.

Dr. Conolly thinks this a mistaken view of the matter. He says the actual condition of pauper lunatics, both in workhouses and private asylums, is entirely overlooked. He says—

"The insane poor are of necessity exposed in both such places to innumerable disadvantages, only to be avoided in larger public asylums. Their diet, their clothing, their lodging, are all generally of the most wretched description; the means of occupation are very limited; space for exercise is wanting; means of recreation and amusement are unthought of or unknown; and security is only effected by confining the limbs of the violent or troublesome, or by buildings so contrived as almost to shut out light and air, and utterly to exclude cheerfulness. All these circumstances are manifestly unfavourable to the recovery, or even to the amendment of those thus confined; and, whilst there is not any foundation for the assertion, that the number of cures, in curable or recent cases, is greater in private licensed houses for paupers than in public asylums, the mortality in such licensed houses has been shown far to exceed that of the public institutions."—Pp. 2, 3.

Dr. Conolly affirms, that of recent cases sent to public asylums, if not epileptic or affected with paralysis, about fifty per cent. recover. This is the average proportion of cures in all the public asylums. This is said to represent the actual curability of insanity, exclusive of relapses. The public asylums at this present moment are absolutely filled with incurable cases, in consequence of the patients having, as Dr. Conolly says, in the first instance been sent in a recent curable state to private licensed houses or to workhouses, and only transferred to public asylums when they became unmanageable or incurable.

Touching the expense of maintaining patients in public and private asylums, Dr. Conolly says that the advantage is on the side of the public institutions; the average charge per week in the private licensed houses being 8*s*. 11½*d*., and in public asylums 7*s*. 6¾*d*., which constitutes a saving of nearly 1*s*. 6*d*. per week for each patient.

The author is at variance with the commissioners in lunacy on the subject of the comparative expense of keeping up public asylums for incurable and curable cases. He says that the views promulgated by the commissioners on this point are fallacious and full of danger. The commissioners say,—

"The great expenses of a lunatic hospital are unnecessary for incurable patients: the medical staff, the number of attendants, the minute classification, and the other requisites of an hospital for the cure of disease, are not required to the same extent. An establishment, therefore, upon a much less expensive scale would be sufficient."—P. 4.

This Dr. Conolly maintains is an unfair view of the matter. It is impossible, in the first place, to separate the curable from the incurable cases in public asylums, and even if such were practicable, it should never be forgotten—

"That a large proportion of the incurable among the insane are even more sensible to all surrounding circumstances than the curable who are labouring under a recent attack; that the whole character of the life of such incurable patients depends on the manner in which they are treated and taken care of; that some of them well know the asylum to be their permanent home; and that most of them, so far from requiring fewer, actually require more means of occupation, more space for exercise, greater opportunities of recreation than the curable, and a greater variety of comfortable arrangements to reconcile them to their situation, and to maintain that habitual content and tranquillity which distinguish a well-regulated asylum from a miserable madhouse." —Pp. 4—5.

On the subject of the plan of building asylums, Dr. Conolly makes many valuable observations. On this point it is not our purpose to enter. We shall merely confine ourselves to a few general observations in connexion with the matter. In constructing asylums, Dr. Conolly says, the first matter to be considered is the character and acquirements of the insane. Hitherto this has been the last point taken into consideration,—the principal object being *security*. Without wishing to under-estimate the importance of security and protection to life, Dr. Conolly conceives that this is quite compatible with cheerfulness, healthy locality, and other internal and external arrangements necessary for the comfort and ease of the poor unfortunate inmates. The asylum should have as little of the asylum, or the idea which is commonly associated with the word asylum, as is consistent with that degree of surveillance that these cases necessarily require. We feel quite convinced that perfect security, and all other requirements necessary to establish health, can be obtained without making our national establishments for the treatment of insanity like prisons and bastiles. The mind of the poor patient, it should never be forgotten, is in a morbid condition—perhaps sinking under the weight of accumulated imaginary misfortune,—borne down to the earth by despair, fancying that he is against all the world, and all the world against him, colouring everything with his diseased fancy. Take such a person to a prison, or an asylum having all the features of one, and what can you expect—a cure? No! an aggravation of the malady under which the party is labouring. It is against this that Dr. Conolly nobly battles, and his position is irresistible.

On the importance of selecting an asylum for the educated classes, Dr. Conolly observes,—

"It should unquestionably be situated amidst scenery calculated to give pleasure to such persons when of sane mind. Those whose faculties have never been cultivated derive little satisfaction from the loveliest aspects of nature, and experience little emotion amidst the grandest. The sun rises and sets, the stars shine and fall, the hills reflect all the variety of brightness and shadow, of wildness and of verdure, and yet are scarcely noticed with more than mere passing attention. But when education has called the higher faculties into life, impressions upon them, even from external nature, become powerful for good or ill, and in the case of a mind diseased, may act as remedies, or aggravate the malady. The celebrated Robert Hall attributed much of his unhappy state of mind, and even his temporary insanity, to a change of residence from a picturesque and interesting part of the country to a cheerless plain, of which the dulness, flatness, and invariable monotony, saddened his heart. Cowper, whose writings indicate exquisite sympathy with the sights and sounds of common rural retirement, and who, like Robert Hall, was occasionally afflicted with insanity, felt awe-struck and overwhelmed when visiting a friend whose house was situated among lofty hills covered with trees. There are few persons of any degree of education much of whose daily and habitual pleasure does not arise from the view of the objects around them; and the first desire of all who can quit the crowd and toil of business, is to be where they can enjoy 'a prospect,' or to surround their houses with shrubs and flowers. Even in the populous city, the pent-up artisan has a bird, to sing to him whilst he works, and a few flowers, which he cultivates with care. We must not neglect such instincts and capacities if we profess to cure diseased minds. Our practice can only securely rest on the consideration of everything, great or little, capable of affecting the mind beneficially or hurtfully."—Pp. 9, 10.

The author objects to a building consisting of more than two stories. He thinks there should be no inhabited attics, and no bed-rooms or dormitories in the basement of the building; all the rooms should be above ground. He considers that great evils have resulted from erecting asylums for the supposed actual number of lunatics within a county. With regard to the architectural form or construction of asylums, he says,—

"I believe there is none so convenient as one in which the main part of the building is in one line; the residence of the chief physician or other officers being in the centre, and also a chapel, and a large square room in which the patients may be occasionally assembled from either side of the asylum on the occasion of an evening entertainment, and which may also be capable of division for schools; the kitchen, laundry, workshops, and various offices, being arranged behind these central buildings. To this main line, wings of moderate extent being added at right angles in each direction, the building assumes what is called the H form; but it is desirable that the length of front should be more extended than that of the wings; and it is better still if the wings only extend in one direction, and away from the front or northward, supposing the front of the asylum to be to the south."—P. 12.

He disapproves of the practice of painting numbers and letters on the walls of airing courts. He says, justly,—

"When it is remembered that many patients are sent to an asylum whose senses are as perfect, and whose feelings are as acute, as those of sane people, and that from the moment they enter the outer gate everything becomes remedial with them, or the reverse, the reason will at once be seen why the external aspect of an asylum should be more cheerful than imposing, more resembling a well-built hospital than a place of seclusion or imprisonment."—Pp. 13, 14.

Dr. Conolly entertains very strong objections against large dormitories. He observes on this subject,—

"Those who sleep in them are generally discontented. The air of such large sleeping-rooms becomes indescribably oppressive when the patients have been two hours in bed; and it never becomes quite fresh and pure, although all the windows and doors are open, in the longest and finest day. One patient, accidentally noisy, disturbs the repose of fourteen or fifteen; and out of that number there is often some one noisy. One man suddenly irritated, or any one patient suddenly starting out of a dream, may rush on his nearest neighbour, and injure him severely. Such accidents are very incidental to dormitories; and in those houses in which they are said to produce no inconvenience, I suspect that all who are likely to be troublesome are fastened to their beds. The violent patients must of course be in single rooms, and if dirty patients are herded together at night, a dormitory becomes perfectly disgusting: and as for the clean, and orderly, and tranquil, and convalescent patients, no complaint is so constantly on their lips as that which arises from their not having a single room, and, consequently, not having a single moment to themselves, or any place where they can be quiet, or, in their frequently uttered words, where they can even say their prayers without interruption. I would therefore have at least two-thirds of the bedrooms single rooms, very few and small dormitories, and no large dormitories for any class of patients."—Pp. 24, 25.

On the subjects of the arrangements of the sleeping rooms, warming and ventilation, lighting, baths, and offensive odours, Dr. Conolly's observations are full of important suggestions. Every word of advice that falls from his pen conveys to the mind of the reader the idea of a man speaking from his own personal knowledge, and feeling the great importance of the truths he is enunciating. His remarks on the above specified subjects are specially applicable to the management and government of public county asylums. Proprietors of asylums for private patients may, however, derive many valuable hints and suggestions from Dr. Conolly's observations. As an illustration of the effects of good management in the Hanwell County Asylum, we may mention, that in one of the largest wards, containing fifty of what are termed dirty patients, many of whom are paralysed, imbecile, and idiotic, all requiring as much attention as infants, there are only four male attendants. This ward is kept in such a cleanly state that there is scarcely ever the least smell in any part of the gallery!

On the subject of restraint, Dr. Conolly enters quite *con amore;* and delightful it is to read the results, noble results, of the non-restraint system of treatment in this large county asylum. Before the introduction of these and other humane views, the poor invalids were placed in small close wards on the third storey at the two extremities of the building, being the wards most difficult of access, and from which the sick could scarcely get out of doors for exercise; placed, too, the most remotely from the chapel, the surgeries, and the kitchen, and even the refractory and dirty wards, with which they communicated by an open staircase. No places, says Dr. Conolly, could have been more unfortunately selected for the sick, less convenient to the medical officers and attendants, and more exposed to disturbance and to bad air. In the days of restraint, says the author,—

"The consequences were, that physical suffering and mental disorder were alike aggravated, and the severest methods of repression resorted to in wards where attention to sickness ought to have been the principal duty of the officers. Violence, noise, refusal of food, destruction of bedding, tearing away of dressings, a disposition to suicide, and all other irregularities, productive of daily agitation, now past and even unknown, but never to be forgotten, were then more frequently witnessed in the infirmaries than anywhere else. I never enter those two wards, now assigned to a different class of patients, without recollecting the miserable struggles, the violence, and the wretched death-bed scenes, characteristic of a time when restraints were so familiarly employed, and so perseveringly kept on, as not even to be removed until life was extinct."—Pp. 46.

The following case is most illustrative:—

"It was in the female infirmary at Hanwell, exactly seven years ago, that I found, among other examples of the forgetfulness of what was due either to the sick or insane, a young woman lying in a crib, bound to the middle of it by a strap round the waist, to the sides of it by the hands, to the foot of it by the ankles, and to the head of it by the neck: she also had her hands in the hard leather terminations of canvas-sleeves; she could not turn, nor lie on her side, nor lift her hand to her face; and her appearance was miserable beyond the power of words to describe. How long she had been in this state it is not material to record. That she was almost always wet and dirty, it is scarcely necessary to say. But the principal point I wish to illustrate by mentioning this case is, that it was a feeble and sick woman who was thus treated. At that very time her whole skin was covered with neglected scabies, and she was suffering all the torture of a large and deep-seated abscess of the breast. Let it be considered what must be the effect on the attendants of having customary recourse to the imposition of restraints, when such complicated suffering as this became comparatively disregarded by medical men, in consequence of the spectacle presented to them being, at each visit, not that of a sick person requiring aid, but of a dangerous lunatic cruelly fastened and bound. But this patient was neither dangerous to herself nor to others. The excuse alleged for this mode of treatment was, that she would eat the poultices employed, and which contained lead, and that she was very mischievous: that was all. However, she was liberated; no bad consequences ensued, and in a few weeks I saw the poor creature at the chapel, and even heard her play the organ, which she had been accustomed to do in the church of a village in Middlesex before her admission. This patient died very recently; having from the time of her liberation from restraints scarcely ever given any trouble to the attendants."—Pp. 47, 48.

Seven years have elapsed since the experiment of non-restraint has been fully tried in the Hanwell asylum, and Dr. Conolly, in the spirit of a Christian philosopher, thanks God, with deep and unfeigned humility, that nothing has occurred during that period to throw discredit on the great principle for which he has so nobly battled.

On the subject of exercise, clothing, &c., Dr. Conolly makes many judicious remarks. We quite agree with him on the importance of active exercise in the open air, considering this a valuable curative agent in the treatment of affections of the mind. In order to obtain the full advantage of out-door exercise, those afflicted with this malady should have free access to rural walks and scenery, flower gardens, groves, &c. Dr. Conolly says—

"No external influence could have been devised more powerful to depress the mind, and sink it into inactivity, than monotonous gravel courts surrounded by walls from ten to fifteen feet high; without a tree; without a shrub; without a blade of grass; without shade in the heat of summer, or shelter from the rains of winter—the only luxury being a bench fastened to the wall, with large iron rings suspended over it, so that even in the open air restraint might still be substituted for superintendence. In these respects, asylums were, until very lately, precisely like jails, in one of which a man, who had been long imprisoned for an act committed in a fit of insanity, used to say to me, when I visited him,—'Sir, I have not seen a flower or a green leaf these seven years!'—a painful observation to the ears of those who can pass from such gloomy precincts to fields and pleasant footpaths, never more to be trodden by the miserable. Within equally melancholy boundaries might be seen, in most asylums, but a few years ago, every form of gloom and eccentricity which the absence of all external objects of interest could foster; and the patients walked up and down some chosen path beneath the hopeless walls, until the very ground was worn into hollows; or, debarred from the full exercise of the muscles of locomotion required by their excited brain and nerves, expended all their energy in exertions of voice, distressing to all within hearing. Even at present, such arrangements may yet be witnessed, and the consequent concentration of morbid excitement of a crowd of insane people, which ought rather to be allowed relief by action and expansion in liberal space."—Pp. 50.

Insane patients should be compelled to take active muscular exercise in the open air, "for nothing has so great a tendency to increase the irritability (of the mind) as keeping patients within doors."

On the diet of the insane, the author gives us the benefit of his enlarged experience, and enforces with great zeal the necessity for generous living. We consider Dr. Conolly's views are so valuable, that we make no apology for quoting his opinions on this point at some length. We do this because many medical men entertain very mistaken notions on this important subject. He says, that with regard to the quality, the quantity, the preparation, and the distribution of the diet of the patients in an asylum, they are subjects in every way worthy of the careful consideration of the managers, the officers, and the attendants. The mere nutrition of the helpless, who cannot express their wants, or represent the most flagrant injustice and privation, demands all the care that humanity can suggest; but it is ordained that man should be capable of associating enjoyments with the mere partaking of food, which communicate satisfaction to the mind; and where the object is the restoration of mental tranquillity, attention to the diet, and its preparation and serving, rank among remedial measures, acting on the mind as well as on the body. All habitual physical discomfort is opposed to mental recovery, and a scanty, ill-cooked, unwholesome diet, creates a chronic uneasiness and dissatisfaction, impairs the health, and increases the mortality of the asylum. There is some reason to suppose that insane patients, shut within the bounds of an asylum, and necessarily leading a monotonous life, require, as prisoners are said to require, a greater quantity of food than persons do who are at large. It seems, at all events, to be established in asylums, that a very low diet conduces to a high mortality, and that the deaths diminish when the diet is improved —facts not difficult of belief, if we remember the number of feeble, paralytic, and phthisical patients in all asylums; and the number sinking from chronic and obscure disease, in all of whom life is capable of remarkable prolongation by careful management. Dr. Charlesworth is, perhaps, even right in considering insanity to be always a disease of debility. There are certainly, at all times, in all asylums, many patients prone to sinking and death, in whom life is only prolonged, and is visibly prolonged, by an extra allowance of diet, with the addition of porter and wine, and sometimes of brandy. The insanity is itself, in certain classes, the frequent result of half starvation, going on for years, or for generations. The body has deteriorated, and the manifestations of the mind fail with the other functions. In some cases, the mere diet and general comfort of the asylum are sufficient, first for relief, and ultimately for cure. Patients who sit down in dismal homes, to poor and ill-prepared food, and to very little even of that, are unacquainted with the meaning of a cheerful meal. Their food is swallowed without pleasure; indigestion follows; disordered conditions of the bowels ensue, and physical and mental ills beyond the apothecary's stores to cure. Removed to an asylum, the sight of good food, in sufficient quantity, surprises them; they sit down to their meals 'free-minded and cheerfully disposed,' one of the 'sure precepts of long-lasting' enumerated by Lord Bacon; they soon become nourished; the body and the mind recover power; and if we do not produce a cure, we produce content, which is a great gain.

"But the cures seem positively to be increased in number by good diet. Before the French Revolution, the diet at the Bicêtre consisted, Pinel says, of a pound and a half of bread daily. This was given out in the morning, and instantly devoured, the rest of the day being passed in a kind of delirious famine, as travellers now report it to be by the patients in the cages of the asylum at Cairo. In 1791, the diet at the Bicêtre was amended, the allowance of bread being increased to two pounds, which was directed to be given in divided portions, with some good soup, morning, noon, and evening. The results of these changes are worthy of remembrance by the directors and officers of public institutions, who are sometimes led into inhumanity, disguised as the respectable virtue of economy. Under the old system, in 1784, out of 110 admissions, there were 57 deaths. After the introduction of the new system, the mortality on the total number admitted was reduced to one-eighth." —Pp. 65—67.

In estimating the proportions of recovered in our public asylums, resulting from an improved dietary and other causes, Dr. Conolly properly observes, that the calculations deduced from the " admissions" are based on a palpable error, for, as he observes—

" If the ultimate fate of every case admitted into an asylum were accurately known, the comparison of the whole number of recoveries with the whole number of admissions would be simple and conclusive; but as this can never be accurately known, the number of admissions does not form a just standard with which to compare the number of known recoveries. If incurable cases are excluded from some asylums and admitted into others, no calculation of cures on the admissions can justly show the curability of insanity in relation to both; and the proportion of recoveries in one must seem unduly greater than in the other. In any one year it is even possible that the recoveries may exceed the admissions. But the average daily number of patients in any asylum forms a fixed standard, in relation to which the average of annual recoveries and deaths may be calculated with at least relative accuracy; and with reference to asylums in which the regulations differ as to the kind of cases admitted.

" In estimating the advantages of different asylums as places of cure, the separate results furnish us, therefore, with no accurate guidance. There is no presumption in saying that Hanwell ranks among the best conducted asylums; yet its general proportion of cures is below that of several others. But Hanwell is the refuge for all the worst cases in Middlesex. Patients are neglected, or fastened down and beaten, in numerous houses in the county, in the recent state of malady, and when rendered intractable and dirty, are sent to us to be cured. Or they are sent to us paralysed, aged, bedridden, deprived of sense, covered with bruises or sores, to die in our infirmaries. Of the first fifteen cases admitted at Hanwell in 1846, one only was recent, or presented a slight prospect of cure, and even this case was one in which the predisposition to insanity was hereditary. Admission is not refused to the epileptic, the paralysed, or the incurable. If they do not recover, they are not sent away. This is their place of rest; here they remain until they die. They consequently fill up the places which might be occupied by the recent and the curable. This is inevitable in a county asylum for paupers only, and it ought not to be otherwise. It precludes Hanwell figuring in a table of comparative cures; but it makes this asylum the home and refuge of those who have no other earthly retreat. Of the recent cases, excluding the paralytic and epileptic, 50 per cent. are found to recover at Hanwell, being about the same number as in asylums where only recent cases are admitted, as Bethlem, St. Luke's, and Liverpool; always remembering, that of these 50, 25 may be considered as liable to relapse."—Pp. 68, 69.

Dr. Conolly does not ascribe these results exclusively to an improved dietary. Patients, prior to admission into Hanwell and other public asylums, have had other great disadvantages to contend with—viz., indifferent lodging, ill-ventilated bed-rooms, scanty clothing, neglect of cleanliness, and often with great harshness, cruelty, and even brutality. It is calculated that the weekly allowance of solid and liquid food allowed to male patients in each county asylum in England is as follows: the quantity varies from 321 ounces of solid and 28 pints of fluid food, to 142 ounces of solid, and 19½ pints of fluid food.*

The following is the present dietary for the pauper patients at Hanwell County Lunatic Asylum, and this, we would premise, is effected at the expense of eightpence per diem, the actual cost of provisions being, *for each patient, four shillings and eightpence halfpenny per week!* But then there are other incidental expenses, which swell the weekly expense of each patient to nine shillings and a penny per week, or 23*l.* 12*s.* 4*d.* per year. The estimate is as follows, calculating from the quarter ending March 31, 1846:—Provisions, 4*s.* 8½*d.* per week; house and bedding expenses, 1*s.* 8¼*d.*; salaries, wages, and maintenance of officers, attendants, and servants, 1*s.* 8¾*d.*; clothing, 8*d.*; medicine and incidental expenses, 3½*d.*; which makes, in the aggregate, the amount above specified.

But to return to the dietary: it is as follows:—

"*Breakfast.*—The breakfast of the men consists of one pint of cocoa, with six ounces of bread; the women have the same quantity of cocoa, and five ounces of bread. The cocoa has only recently been substituted for milk-porridge, to the great satisfaction of all the patients.

"*Dinner.*—On Sundays, Tuesdays, Wednesdays, and Fridays, the dinner for each patient is five ounces of cooked meat, (seven ounces uncooked,) four ounces of yeast dumpling, and twelve ounces of vegetables. The meat is cooked by steam, except on Sundays, when it is baked, making the Sunday dinner a welcome variety. On Mondays the dinner consists of one pint of soup, with six ounces of bread; but as many patients dislike soup, eleven ounces of currant dumpling are occasionally substituted for it, and this seems to be approved of by the patients. On Thursdays, the dinner consists of twelve ounces of Irish stew, (containing an ounce and a half of cooked meat,) with six ounces of bread; and on Saturdays, of twelve ounces of meat-and-potato pie, containing about an ounce and a half of meat, two ounces being weighed out for each patient before cooking. Each patient has half a pint of beer at dinner-time. In the fruit season, fruit pies are sometimes substituted for the meat pie on Saturday. Occasionally the patients have bacon and beans for dinner, and green vegetables are frequently substituted for potatoes. I believe all these varieties are as salutary as they are acceptable to the patients. The chief inconvenience of any fixed dietary is a want of variety; change of diet seeming to be desired, and even required, by patients of every class.

"*Tea.*—The female patients alone are allowed tea in the afternoon; one pint of tea, with five ounces of bread, and half an ounce of butter, constituting at once their tea and supper. Many of the male patients feel the loss of their tea as a great privation when newly admitted. The friends of the patients are allowed to send them tea and sugar. The tea is prepared in the kitchen, by steam, and, probably from the extraction

* Dr. Begley.

of the bitter principle of it, is not generally approved of; but in so large an asylum, making tea in the wards is scarcely practicable.

"*Supper.*—The male patients have two ounces of cheese, six ounces of bread, and half a pint of beer, at seven in the evening.

"*Extras.*—The men who work in the gardens, and on the farm, have half a pint of beer at eleven and at four o'clock, and are also allowed one ounce of tea and four ounces of sugar per week. The women who work in the laundry are allowed half a pint of beer at eleven o'clock, with bread and cheese. The elderly and feeble women in the ward No. 2, have a meat dinner daily. Those in the infirmaries, also, unless meat is improper for them, have a daily meat dinner. Fish, or some substitute for meat, is allowed on Fridays, and in Lent, to the Roman-catholic patients; and the sick have whatever the medical officers think it right to order for them, and are supplied, during the night also, with tea, coffee, beef-tea, sago, and arrow-root, with or without brandy, &c. &c.; and by these attentions they are kept from sinking, and the tedious length of the night hours is broken."—Pp. 70—72.

The meat is of the first quality, and great attention is paid to cleanliness, order, and punctuality. The attendants do not dine with the patients, but merely wait at table. Grace is said before and after meals. The patients, with few exceptions, have the use, during meals, of knives and forks: the knife is sharpened along a portion of its edge. The patients are less destructively disposed, and crockery has been lately introduced into the wards. The breakage is quite insignificant. A nickel fork is used by the patients, and is shaped like an ordinary silver one. We mention these particulars to show how carefully every minute matter involving the comfort, happiness, cure, and safety of the patients in this asylum is attended to by those in authority. If it be necessary for the treatment of pauper patients that such especial attention should be paid to these points, *à fortiori*, how absolutely indispensable is it that patients of a higher class, confined in private asylums, should have around them all the comforts and little elegancies of life to which they have been accustomed when well, and whilst at home. As Dr. Conolly says:—

"It is impossible to be too careful in directing that all the service of the table should be in accordance with the habits of the patients. The sense of banishment from home, and of confinement, and the consciousness of mental infirmity and dependence, are mitigated in the mind of many a silent, uncomplaining patient, by these means. All the comforts added of late years, and with so much advantage, to the wards of county asylums—such as moveable tables and chairs, window-blinds, plants, musical instruments, bagatelle-boards, books, and pictures, with free access to agreeable gardens—show that the majority of the insane of any class are inclined to respect the decent arrangements made for them; and it will generally be found, that the more comformable the furniture of the rooms, the tables, &c., of the higher class of the insane is to their habits and rank, the less they will be disposed to destroy or derange it. During violent paroxysms of mania, they of course exhibit great recklessness; and everything about them should be plain, simple, and secure. But when convalescence begins, it should be respected. Among the depressing recollections of the insane of the higher classes, when recovering from insanity, I know that none are more frequent, or felt to be more degrading, than those connected with any want of respect shown to them, or any disregard of decent customs as to their meals. Yet, without great attention, they will sometimes be found, when quite well enough to appreciate what is done, sitting down to a dinner of meat, vegetables, and pudding, all sent to them on one plate. Negligences of this kind produce fretfulness and discontent, and tend to retard convalescence. If attendants are allowed to practise this kind of negligence, they soon fall into habits of rudeness, and even of inhumanity, fancying that the patients do not observe their conduct, and that their feelings are of no consequence."—Pp. 76, 77.

On the subject of "employment," it is gratifying to find, by the last annual report, that of 418 male patients then in the asylum, 219 were employed; of these 75 were occupied in the gardens, or in the farm, and 40 as helpers in the ward. There exists at Hanwell a carpenter's, a shoemaker's, and tinman's shop. The patients are engaged as bricklayers, whitewashers; some in the engine-house, the smith's shop, gas-house, printing-office, &c. Of 357 women, 314 were employed; 69 in the laundry, 49 as helpers in the wards, 18 in the kitchen and dairy, and 178 at needlework.

We approach now the consideration of what we conceive to be an important, if not one of the most important, parts of the economy of an establishment, be it private or public in its character. We refer to those who are employed in such institutions as attendants on the insane. Imprimis, we have a strong objection to the term "keeper," an appellation still used in some asylums. We consider this a most offensive designation, and one only calculated to originate in the mind ideas peculiarly repugnant to the feelings, whether they be sound, or in a morbid state. The word "attendant" is the common and generally received name applied to those who act in the capacity of servants in asylums. On many occasions, where the case is peculiar in its character, and there exists an unnatural amount of morbid sensitiveness, we have often substituted the word *servant* for attendant, with the happiest result. No attendant accustomed to the management of the insane, and whose feelings qualify him for such anxious and onerous duties, will object to be addressed by such an appellation. They ought, in fact, to be considered as the servants of those placed under their care; exercising, of course, a discretionary power in obeying the mandates of those of whom they have charge. The post of an attendant is a most responsible and anxious one. Their tempers are often severely tried; they are compelled frequently to submit to great personal abuse,—are liable to be kicked, cuffed, spat at, and occasionally foully abused by those who conceive themselves to be most unjustly deprived of liberty, and who therefore feel authorized to adopt any means within their power to resent an imaginary illegal interference with their free agency. Then, again, the conduct of attendants is liable to great misapprehension; often the most cunningly devised tales are concocted against them by the patients. Stories having every semblance of truth are told to the medical superintendents and commissioners in lunacy, with reference to particular alleged acts of barbarity and cruelty. We mention these particulars to establish, that much is to be said for those who act in this capacity,—that their duties are not the most enviable in their nature, and that every reasonable indulgence ought to be allowed them. They must necessarily have great command of temper, for not the remotest semblance of a shade of retaliation would be allowed. It requires something more than great self-possession to stand with impunity the abuse often levied most unsparingly against them. We always, under these circumstances, reason with the attendants, by letting them understand that they have the management of persons deprived of a proper use of reason,—that in many cases all self-control is lost,—in fact, that whilst insane, they are not responsible for their actions,—and that any cruelty, harshness, or the appearance of unkindness, even by a look, will not be for one moment tolerated. We feel it is quite necessary to exercise military law occasionally, and to forbid certain deviations from regular conduct, on pain of instant dismissal and disgrace. We feel, with Dr. Conolly, that good attendants make good and manageable patients; and that they have it in their power to promote or retard the recovery of those they have the care of. The attendants, even the best of them, require constant and vigilant superintendence. With every disposition to be kind, attentive, and courteous in their behaviour, they often, for want of a stimulus, relax in their duties; hence the importance of having always upon them the eye of a person acquainted with their duties, and with resolution to insist upon their most rigid performance. In public asylums, the proportion of attendants is one to seventeen patients; Dr. Conolly thinks it ought to be one to fifteen. In French asylums, the proportion is one to ten. In private asylums, the proportion should be one to five, or one to three. Occasionally, patients require the exclusive attention of one or two attendants, and their friends expect this, and in some cases it is absolutely necessary. In the choice of attendants, Dr. Conolly observes:—"No subject connected with the management of the insane, either in asylums or in private practice, has received less adequate attention than the selection of proper attendants, their proper treatment, their just government, and their instruction in the various, and peculiar, and exhausting duties which necessarily devolve upon them. The important and delicate task of regulating the conduct of persons of unsound mind, of controlling excitement, restraining waywardness, or removing mental depression, is unavoidably confided to persons of limited education; but these are too frequently chosen with little regard to their disposition, temper, or intelligence: they are permitted to commence their duties with as little preparation as if their office was merely that of a servant, and are governed either with severity and injustice, or without the consideration and indulgence requisite to support their patience, and to encourage them to be considerate and indulgent to those on whom they attend, and who are wholly in their power."—P. 83.

Again the author observes:—"The character of particular patients, and of all the patients of a ward, takes its colour from the character of the attendants placed in it. On their being proper or improper instruments,—well or ill-trained,—well or ill-disciplined,—well or ill-cared for,—it depends whether many of his patients shall be cured or not cured; whether some shall live or die; whether frightful accidents, an increased mortality, incalculable uneasiness and suffering, and occasional suicides, shall take place or not. Painful, therefore, is the situation of the physician to an asylum who is liable, at every visit, to find forty or fifty of his patients put under the care of new attendants; separated from those to whom they were attached, and who had become acquainted with their peculiarities; and subjected to the rude dominion either of attendants without experience, and whom he sees at once to be without capacity for the duties he requires of them, or, what is worse, of attendants trained in asylums where the system of restraint is in full operation, and who are consequently versed in all the neglect, the harshness, and the concealment which can inflict suffering on the patients, and which must cause disappointment of all his designs."—P. 84.

On the choice of attendants, and the qualifications necessary, Dr. Conolly speaks like a man of great experience:—

"As a general rule, the attendants, when entering on their duties, should not be more than thirty years of age, or rather, five and twenty. Male attendants, who are older, if they have been in the army, or accustomed to responsibility, often prove valuable; but I have scarcely ever known a female attendant prove efficient who commenced her duties after thirty, and some of the best whom I have known, and whom many years of trial have now proved, began their duties before they were twenty. Activity and good spirits are required, and these qualities do not increase with years. The temper of older persons unaccustomed to the insane is easily ruffled; they have often been depressed by the events of life, and either give way to the oppressive influences of an asylum, or try to keep up their energies by stimulants. This is particularly the case with female attendants who enter late on such duties; and altogether—and putting humanity as much out of the question as it generally is where attendants are selected in the manner I have mentioned—there is no cherished fallacy greater than that of supposing that plain, middle-aged, ill-dressed women are more moral, or more orderly in their habits of life, than young, active, well-dressed nurses. The best attendants, both male and female, are to be found in the class of persons who are qualified to be upper servants, and their services may always be commanded in an asylum where a fair remuneration and a prospect of comfort are held out to them.

"But the first requisite for an attendant, if conjoined with a moderate share of understanding, is benevolence I fear this is a quality often wholly omitted in the inquiry into the character of attendants. Certainly, many have been observed by me in such situations, and entrusted with the care of private patients, whose physiognomy and whose voices proclaimed at once their want of such a quality; men chosen apparently on account of their possessing the frame of a prize-fighter, and sharp-tempered women, with merely capacity enough to be under-housemaids. Yet these attendants constantly live with the patients, and must be entirely relied upon for the performance of the various duties which are presently to be mentioned. There is generally of course, in asylums, a rapid succession of attendants so ill qualified as these; and sometimes for a whole year, or for successive years, some particular wards are thus kept continually unsettled and ill-managed. Irregularities, accidents, injuries, and confusion then present themselves daily to the physician's observation, and he is powerless to remove them. It cannot be concealed or denied that where such regulations prevail, and such attendants are appointed, the patients have not all the advantages which the public have a right to expect should be extended to them, and that a great duty is neglected. The great object of establishing and perpetuating a system of kindness, which nothing can disturb, and which generates the confidence of the patients, and acts as the most powerful and salutary of all restraints, is thus defeated.

"It is quite impossible for me to convey to the reader a just idea of the mischievous cruelty exercised in private houses, where single patients are entrusted to attendants supplied from private asylums in which restraints are permitted to be used. They invariably arrive armed with a strait waistcoat; they almost invariably put it on the patient, whose subsequent violence becomes an excuse for drawing it closer and tighter, until the skin is ulcerated. The legs are then fastened, and the legs and back become ulcerated also. Cleanliness is then disregarded; food is refused in some instances after such degrading treatment, and death, I sincerely believe, is an occasional result." —Pp. 85—87.

The author's observations on the duties of attendants are so valuable, that we make no apology for quoting a passage of some length from his work:—

"Attendants should be accustomed habitually to watch for the first signs of recovery, and to promote the progress that they see beginning. The indications of this, in an altered language, or dress, or manner, if neglected, may pass away for ever. Patients at this critical period require most delicate attention. Recollections which were lost in the past excitement, come back irregularly, and with them, past feelings, as a remembered dream. Severe fits of sobbing and crying are then usual in female patients, and broken expressions relative to their past history, their errors, their griefs, their home, and those who loved them. They are, at this time, particularly sensible to kind words, but easily disturbed by violent impressions or painful emotions; and it is now, when an indiscreet word may retard recovery, that all the physician's trust, and all his hope, rest on the judicious aid of kind attendants.

"These duties can only be reasonably expected from attendants of humanity and intelligence, who are treated kindly, governed justly and mercifully, and properly supported by the officers. If they look with dread to a passionate manner and words, which they expect to be manifested or addressed to them when the door of their ward is opened by an officer of the asylum, and are subjected to have degrading rebukes addressed to them, and threats, in the hearing of the patients, not only must their useful influence be lessened, but they must become disgusted with their duties, and sullen, and negligent, and too prone to direct some portion of their irritated feelings towards the patients, who have been already disturbed by the imperious tones and gestures of some officer, whose temper is unfitted to bear the unavoidable agitations and irritations of an asylum. The morning or evening visit of every officer should be the visit of a friend, alarming to none, unwelcome to none who endeavour to do their duty. It then becomes a highly useful means, of daily application, not always producing immediate results, but greatly influencing the character of the wards, and of the house, by daily repetition. These visits should be considered by the officers as the first and chief object of the day. If they are postponed until many other matters have been attended to, the officers run the risk of entering the wards with a discomposed temper, and consequently in a humour to discompose the attendants. Every inquiry should be made, and every order given, with calmness. Cruelty and neglect alone should be at once and openly reprimanded, yet not without some moderation. Deserving attendants will then cheerfully and truthfully communicate to the officers all that has passed in the ward, or all that they know; will represent any particular difficulties, sure of being aided in combating them; and will feel a gratification in pointing out any trifling change in any patient which indicates the first returning gleams of reason. Attendants who zealously endeavour to perform all their duties well should be treated with confidence, and allowed every reasonable indulgence. The natural pride they feel in the state of their ward, and in their influence over troublesome patients, should not be discouraged by frequent removals either of the patients or of themselves from one ward to another. Everything that affects them will be found to exercise a secondary influence on the patients; their health, their circumstances, their instruction, their mode of living in the asylum, their diet, the comfort of their sleeping-rooms, are all matters of much importance, generally attracting too little attention, and sometimes wholly overlooked in the arrangements of an asylum. Yet nothing can be more certain, than that attendants who lead an uncomfortable life are not likely to be in a state of mind to make patients comfortable. If it is forgotten that they have ordinary human feelings, or if they are treated as if presumed to be always dishonest and unworthy of trust, or are subjected to heartless or capricious refusals and mortifications when desirous of a little extra leave of absence, they cannot but lose all attachment to the officers, and all interest in the asylum. It is a mockery to tell attendants, who are not sure of their places for a day, that they are to devote themselves entirely to the patients, and to be themselves patterns of forbearance. Yet the want of devotedness on their parts is a want of that for which there is no substitute, and the want of forbearance in the attendants will lead to the worst consequences. So strictly connected is the proper government of an asylum with the welfare, and even with the safety of the patients. Unjust officers, or unjust rulers, make attendants indifferent or cruel, and indifferent or cruel attendants make the patients wretched.

"When I had authority over the attendants at Hanwell, it was a rule with me never summarily to dismiss an attendant, except for cruelty; but never to overlook cruelty. There is no other security for the patients. Where a physician does not possess authority to do this, or any asylum is governed with slight regard to his general views, or other officers have authority quite independent of him, other faults will be punished by dismissal; but cruel conduct, the worst of all faults, will too often appear to be considered venial, and the attendants will too soon learn that common humanity may be disregarded, and the medical officers defied with impunity. The ignominious dismissal of an attendant on the insane is often a sentence of destitution; for unless they can meet with similar employment, they can seldom find any employment at all: it should therefore, on this account also, be reserved for cruelty or some serious offence alone; but for cruelty no excuse should be permitted. I should not dwell so much on this point of discipline if I did not believe it to be often incredibly undervalued, and with grievous effects on the patients.

"It should be a rule that the attendants should never use violent or intemperate language; should never venture to strike or ill-treat a patient, or to employ the term punishment in relation to anything done to them. They should not even talk to the patients in a loud and scolding manner, nor give directions or collect the patients for dinner, or work, or exercise, or prayers with shouts and disturbance; nor should they persevere in arguing with them, or contradicting them, or reproaching them for their faults. They should be always vigilant, should seldom interfere, but be ready for prompt interference when necessary. The general character of each ward should be tranquil, and yet the superintendence by eye and ear so perfect, that no person should pass through the ward, and no door be unlocked, without immediately attracting the attention, and occasioning the approach, of an attendant.

"For protection in cases of great violence, the attendants must be entrusted with the power to seclude a patient; but the seclusion must always be immediately reported. The seclusion itself, which is merely putting the patient out of the gallery, or airing-court, into his bed-room, or into a padded room, and locking the door, should be effected without violence; and in cases of difficulty, by the united and prompt aid of many attendants, by whose conjoined exertions danger is best prevented, and the patient best overcome, without any doubtful, dangerous, and irritating contest between the patient and any one or two attendants. It may often be effected by persuasion alone, the patient having generally some consciousness of the desirableness of being quiet and alone. When the use of the strait-waistcoat, and all other means of restraint was first forbidden at Hanwell, seclusion was resorted to for the protection of the attendants, and also because it best screened the patient from external excitement. It is now itself rarely resorted to, except as a remedial measure, and long seclusions have vanished with the wild confusion that prevailed when every excited patient was bound up in hard canvas and leather, or fastened in a coercion-chair.

"The principle of promptly assisting one another should be rigidly adhered to by the attendants of an asylum. When the whistle, with which each attendant is provided, is blown, all within hearing should fly to the point from whence the signal proceeds, and every existing difficulty will then soon be overcome. The attendants are often averse to using this signal, and will encounter patients single-handed—a practice objectionable and dangerous, and which explains, without excusing, many injuries incurred both by patients and attendants. In such single contests somebody must be hurt. A strong array of attendants generally prevents resistance, and always makes it more safely as well as more easily overcome. But when a violent patient has been put into seclusion, or made to take a bath, or to dress or undress, by the united exertions of several attendants, attempts must afterward be made to conciliate him, and restore him to his self-esteem; and this will not be difficult when he has only yielded to numbers, who have merely overpowered him, or locked him up without hurting him."—Pp. 111—116.

The concluding portion of Dr. Conolly's treatise has relation to "religious services," "officers," and "superintendents." Upon all these important subjects he enters fully. We must reserve our analysis of this portion of the volume for another occasion, when we hope to enter minutely into its consideration. Dr. Conolly's book ought to be read by every person having anything to do with the treatment of insanity. Its pages are replete with wisdom, humanity, and love of his fellow-men.

